# Patient characteristics and determinants of CD4 at diagnosis of HIV in Mexico from 2008 to 2017: a 10-year population-based study

**DOI:** 10.1186/s12981-021-00409-0

**Published:** 2021-11-13

**Authors:** Amilcar Azamar-Alonso, Sergio A. Bautista-Arredondo, Fiona Smaill, Lawrence Mbuagbaw, Andrew P. Costa, Jean-Eric Tarride

**Affiliations:** 1 Department of Health Research Methods, Evidence, and Impact (HEI), Faculty of Health Sciences, McMaster University, CRL 201, 1280 Main St West, Hamilton, ON L8S 4K1 Canada; 2 Gilead Sciences Mexico S. de R.L. de C.V, Mexico, USA; 3 Center for Health Systems Research, National Institute of Public Health, Mexico, USA; 4 ChB Department of Pathology and Molecular Medicine, Faculty of Health Sciences, McMaster University, Hamilton, ON Canada; 5 Center for Health Economics and Policy Analysis (CHEPA), McMaster University, Hamilton, ON Canada; 6 Biostatistics Unit, Father Sean O’Sullivan Research Centre, St Joseph’s Healthcare, Hamilton, ON Canada; 7 Department of Medicine, McMaster University, Hamilton, Canada; 8 Programs for Assessment of Technology in Health (PATH), The Research Institute of St. Joe’s Hamilton, St. Joseph’s Healthcare Hamilton, Hamilton, Canada

**Keywords:** HIV, Late diagnosis, Mexican *SALVAR*

## Abstract

**Background:**

In 2007–2012 the Mexican government launched the National HIV program and there was a major change in HIV policies implemented in 2013–2018, when efforts focused on prevention, increase in early diagnosis and timely treatment. Still, late HIV diagnosis is a major concern in Mexico due to its association with the development of AIDS development and mortality. Thus, the objectives of this study were to identify the determinants of late HIV diagnosis (i.e. CD4 count less than 200 cells/mm^3^) in Mexico from 2008 to 2017 and to evaluate the impact of the 2013–2017 National HIV program.

**Methods:**

Using patient level data from the SALVAR database, which includes 64% of the population receiving HIV care in Mexico, an adjusted logistic model was conducted. Main study outcomes were HIV late diagnosis which was defined as CD4 count less than 200 cells/mm^3^ at diagnosis.

**Results:**

The study included 106,830 individuals newly diagnosed with HIV and treated in Mexican public health facilities between 2008 and 2017 (mean age: 33 years old, 80% male). HIV late diagnosis decreased from 45 to 43% (P < 0.001) between 2008 and 2012 and 2013–2017 (i.e. before and after the implementation of the 2013–2017 policy). Multivariable logistic regressions indicated that being diagnosed between 2013 and 2017 (odds ratio [OR] = 0.96 [95% Confidence interval [CI] [0.93, 0.98]) or in health facilities specialized in HIV care (OR = 0.64 [95% CI 0.60, 0.69]) was associated with early diagnosis. Being male, older than 29 years old, diagnosed in Central East, the South region of Mexico or in high-marginalized locality increased the odds of a late diagnosis.

**Conclusions:**

The results of this study indicate that the 2013–2017 National HIV program in Mexico has been marginally successful in decreasing the proportion of individuals with late HIV diagnosis in Mexico. We identified several predictors of late diagnosis which could help establishing health policies. The main determinants for late diagnosis were being male, older than 29 years old, and being diagnosed in a Hospital or National Institute.

**Supplementary Information:**

The online version contains supplementary material available at 10.1186/s12981-021-00409-0.

## Background

Worldwide, more than 37.9 million people are living with HIV (PLWH) of which it was estimated that around 230,000 live in Mexico but only 79% know their status [1–3]. In Mexico and elsewhere, the number of individuals diagnosed with HIV has increased during the last decade due to more transmission as well as disease awareness and the implementation of programs aimed to identify people at the early stage of the disease [4]. In 2019, approximately 17,000 Mexicans were diagnosed with HIV, most of them were male (85%), between the ages of 25–39 years (70%) and contracted HIV due to sexual contact (71%) [5]. Recognizing the problem as a public health matter, the Mexican government established twenty years ago different programs to improve HIV national indicators and the overall health of PLWH. In the beginning, efforts were focused on prevention for high risk populations [6], and the implementation of rapid testing aimed at pregnant women [2]. An important improvement in HIV policies happened in 2003 when the Mexican government expanded the coverage of HIV treatment and care in public health facilities to people who were unemployed and individuals from the informal sector [7, 8]. In 2007, the National HIV program targeting prevention of HIV transmission in key high-risk populations -e.g. men who have sex with men, sexual workers- was launched and was in effect for 5 years [2, 6]. A major change in HIV policies in Mexico was observed in 2013 when the 2013–2018 National HIV program was implemented with the mandate to expand efforts beyond the prevention of HIV transmission among high-risk populations to include populations at a lower risk (e.g. young people and women) and to increase early diagnosis and timely treatment [9]. In 2014, the Mexican clinical guidelines were changed to recommend ART initiation regardless of baseline CD4 count and symptoms [9, 10]. One of the key objectives of the 2013–2018 National HIV program was to decrease the percentage of individuals diagnosed with late HIV to 30% in 2017. With prevalence rates reported of 61% between 2001 and 2008 [11] and 49% between 2008 and 2013 [12], late HIV diagnosis is a major public issue due to its association with the development of complications (e.g. AIDS) and mortality [11–13]. To support these efforts, the HIV budget also doubled and additional resources were allocated to improve HIV care across Mexico [14, 15]. For example, the number of HIV facilities outside of Mexico City increased from 49 in 2007 to 137 in 2017 [4, 16].

Timely diagnosis and initiation of antiretroviral therapy are crucial to ensure optimal health outcomes among PLWH [17]. However very few studies have been conducted to evaluate the impact of Mexican HIV policies on outcomes. For example, one study reported that in 2012 (5 years after the implementation of 2007–2012 HIV national program), more than half of patients died within six months after diagnosis, and the main factor associated with death was a late diagnosis [18]. More recent government reports have documented that the percentage of individuals diagnosed with less than 200 CD4 cells/mm^3^ decreased from 50 to 40% between 2015 and 2018 [19, 20], suggesting a positive impact of the HIV policy in Mexico. However, only one study has examined the predictors of late diagnosis in Mexico using data from 2007 to 2014. While results indicate that men and older adults (more than 50 years old) were at a higher likelihood of late diagnosis [17] compared to the general population, this situation may have changed following the modifications in HIV policies that happened in Mexico after 2013. To better identify people at high-risk for a late diagnosis, and to inform further policies and strategies in Mexico to reach these people early, the main objectives of this study were to identify the determinants of late HIV diagnosis in Mexico between 2008 and 2017 and to evaluate the impact of the 2013–2017 National HIV Program.

## Methods

### Study design

The design was a retrospective population-based cohort study using Mexican administrative health data from the antiretroviral therapy administration, logistics, and surveillance system (*Sistema de Administración, Logística y Vigilancia de Antirretrovirales—SALVAR* for its acronym in Spanish).

### Data source

The *SALVAR* database is an electronic system created in 2006 by the National Center for the Prevention and Care of HIV/AIDS to manage the clinical information of PLWH enrolled in the HIV/AIDS program led by the Mexican Ministry of Health (approximately 64% of all PLWHs in Mexico). While *SALVAR* was developed in 2006, it was not until 2008 that it was operational across Mexico. Currently, the system contains linked information on more than 172,000 Mexicans living with HIV (e.g. baseline characteristics, treatment regimens) receiving treatment [20].

### Study population

The study population includes adults (18 years or older) diagnosed with HIV between January 1st, 2008, and December 31st of 2017 in health facilities affiliated to the Ministry of Health. Children, people who are incarcerated, and people receiving antiretroviral prophylaxis were excluded from the analyses. Individuals with incomplete information on gender, age, date of diagnosis, and results of the first measurement of Viral Load (VL) and CD4 were also excluded.

### Study outcomes

The primary outcome of the study was HIV late diagnosis, which was defined as CD4 count less than 200 cells/mm^3^ closest to the date of diagnosis reported in *SALVAR* [21]. As the dataset does not specifically contains a variable for CD4 at moment of diagnosis, it was considered that the measurement closest to the date of diagnosis (or with the same date), was the key measurement. Secondary outcomes included CD4 cell count at time of diagnosis, CD4 cell count stratified based on WHO recommendations: less than ( <) 200, 200–349, 350–499 and, equal or more than ( ≥) 500 cells/mm^3^ [22], and VL at time of diagnosis stratified as VL ≤ 100,000 units by milliliters of blood (u/ml) and more than 100,000 u/ml [23] for descriptive proposes. For CD4, a lower limit of zero and an upper limit of 2,000 cells/mm^3^ was established based on clinical literature [17, 24, 25].

### Independent variables

Age was described as a continuous variable reporting the mean and median, also age was categorized in groups (18–29, 30–39, 40–49, 50–59, and ≥ 60 years old). Gender was stratified as male, female, and transgender. Characteristics related to health facilities were also included. For the purpose of the study, Mexico was divided into five regions [18, 26] (Central West, Central East, Northwest, Northeast, and South) and Mexico City was also counted as an additional region due its large population (25% of Mexicans live in Mexico City) and distinct structural characteristics. In addition, marginalization indices [11, 17] grouped into three categories (high/ very high, medium and very low/low) were used to capture social and economic differences by locality of the health facilities where care is provided. Health facilities in which diagnosis and care are provided were described in terms of (1) Hospitals and National Institutes that provides primary and specialty care in a tertiary level hospital; (2) Ambulatory Centers for Prevention and Attention for HIV/AIDS and Sexually Transmitted Infections (*CAPASITS* in Spanish) facilities which are specialized, stand-alone centers that provide ambulatory care for HIV and STI; and (3) Condesa, a specialized clinic for HIV located in Mexico city and which provides HIV ambulatory care for more than 15,000 PLWH in Mexico City.

### Statistical analysis

Key characteristics of the study population were divided and compared in two periods according to policies changes, between 2008–2012 and 2013–2017. Student t-test and Welch test were presented for normally and non-normally distributed continuous variables while Chi-square tests were used for categorical variables. Due to expected skewness of some variables, mean values along with standard deviations (SDs) as well as median values and interquartile ranges (IQR) were used to summarize continuous variables (e.g. age, CD4 levels). Discrete variables were presented using percentages. Patients’ and healthcare facilities’ characteristics as well as outcomes (i.e. CD4 and VL) were presented for the periods of analysis to illustrate trends over time.

Logistic regressions were conducted to identify the determinants of late HIV diagnosis (i.e., CD4 < 200 cells/mm^3^). All models were adjusted by the covariates mentioned above. In addition, a dummy variable corresponding to the period when diagnosed (2008–2012; and 2013–2017) was used to estimate the impact of the 2013–2018 HIV policies implemented in Mexico. To have a better understanding of the determinants of late HIV diagnosis before and after the implementation of the National HIV program in 2013, separate models were estimated for the period 2008–2012 and 2013–2017. Logistic models were reported using Odds Ratios (OR) and associated confidence interval. Models’ goodness of fit was evaluated with Receiver Operating Characteristic (ROC) curves and C-statistic [27], where a C-statistic value of 0.70 or greater indicates good discrimination.

## Results

### Study population characteristics

**Fig. 1:**
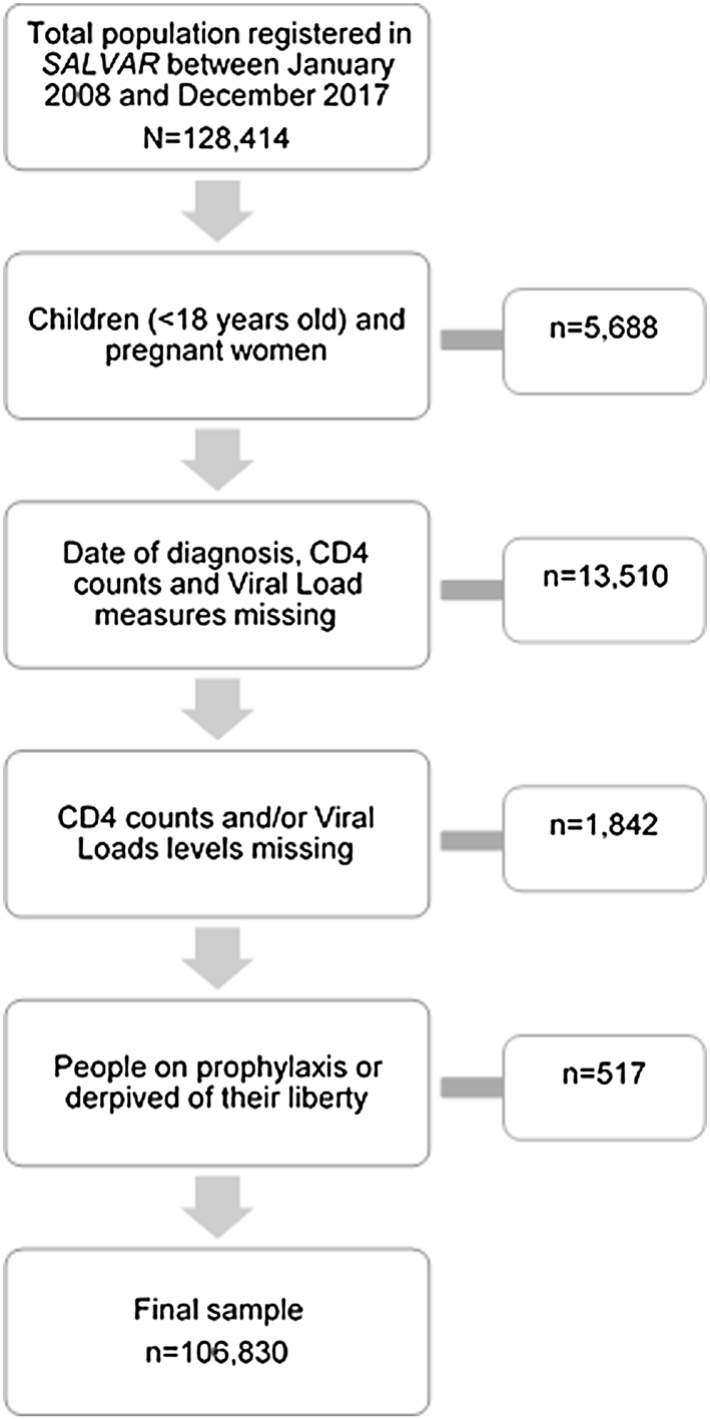
Sample flow for analysis. SALVAR: *Sistema de Administración, Logística y Vigilancia de Antirretrovirales - SALVAR *for its acronym in Spanish

From the initial 128,387 individuals included in the *SALVAR* database for the period 2008–2017, 4,759 were children, 13,802 did not have information on diagnosis, CD4 counts or VL results’ date, 2,254 patients did not have information about CD4 and VL, 23 people were on prophylaxis, and 531 were people deprived of their liberty. After excluding these groups (Fig. 1), the study population consisted of 107,018 individuals. Compared to individuals with complete data, individuals with missing data were mostly males (79.8% vs 60.15%, p < 0.001) diagnosed in the Northwest region (13.3% vs 11.8%, p < 0.001), in CONDESA (33.2% vs 13.57%, p < 0.001), and after 2013 (77.8% vs 55.3%, p < 0.001).

Table 1 summarizes the patients’ characteristics at diagnosis over the period 2008–2017 and for each of the two periods of the analysis (2008–2012 and 2013–2017). The median age of the population was 31 years old (IQR = 25, 39) and 80% were males. Almost two thirds of the study population were diagnosed in *CAPASITS* facilities, one third in the Central East region and 94% in regions with low marginality index (less deprived areas). Most of the health facilities were in regions of low marginalization index. Several changes were observed over the two periods of analysis (i.e. 2008–2012 and 2013–2017). For example, the median age decreased from 32 (IQR: 26, 40) to 31 (IQR: 25, 39) years old between 2008–2012 and 2013–2017 (*P* < *0.001*) while the proportion of individuals aged 18 to 29 years of age at diagnosis increased from 40% in the period 2008–2012 to 45% in 2013–2017 (*P* < *0.001*). During the same period, the proportion of male individuals at diagnosis increased from 77 to 81% (*P* < *0.001*). More individuals were diagnosed in the South of Mexico over time (*P* < *0.001*) and less in the Northwest region (*P* < *0.001*). Table 1 provides the details.

**Table 1:** Summary statistics and patients’ characteristics, CD4 and viral load at diagnosis at diagnosis, 2008–2017

	Total	2008–2012	2013–2017	*P*-value
Sample	107,018	47,702 (44.58%)	59,313 (55.42%)	
Age				
Mean (SD)	33.09 (10.46)	33.50 (10.34)	32.53 (10.50)	< 0.001
Median (IQR)%	31 (25, 39)	32 (26, 40)	31 (25, 39)	
18–29 yo	43.28	40.04	45.72	< 0.001
30–39 yo	32.12	34.38	30.39	
40–49 yo	16.77	17.88	15.94	
50–59 yo	6.03	5.92	6.14	
≥ 60 yo	1.8	1.79	1.81	
Gender (%)				
Male	79.80	77.78	81.4	< 0.001
Female	19.79	21.82	18.16	
Transgender	0.41	0.40	0.41	
Health facility (%)				
Hospital/ National Institute	20.59	21.12	19.38	< 0.001
CAPASITS	66.46	66.32	66.31	
Specialized clinics	13.1	12.57	14.31	
Region (%)				
Mexico City	16.85	16.68	17.27	< 0.001
Central East	30.89	31.60	31.14	
Central West	13.10	13.61	13.18	
Northeast	13.10	13.89	11.84	
Northwest	8.49	8.61	8.39	
South	16.85	15.61	18.17	
Marginality Index (%)				
High	2.05	1.85	2.21	< 0.001
Medium	1.20	1.13	1.26	
Low	93.61	97.02	96.53	
CD4 cells				
Mean (SD)	289 (254)	286 (256)	291 (252)	< 0.001
Median (IQR)	233 (88, 417)	226 (88, 410)	238 (89, 422)	< 0.001
* %*				
< 200	44.93	45.58	43.8	< 0.001
200–349	22.47	22.44	22.36	
350–499	15.91	14.84	16.01	
≥ 500	17.79	17.14	17.83	
Viral Load (log10)				
Mean (SD)	4.28 (1.39)	4.15 (1.42)	4.38 (1.36)	< 0.001
Median (IQR)	4.67 (3.56, 5.27)	4.59 (3.13, 5.19)	4.73 (3.80, 5.32)	
* %*				
≤ 100,000 u/ml	62.72	64.76	61.35	< 0.001
> 100,000 u/ml	37.44	35.24	38.65	

*SD* Standard Deviation. *Yo* years old. *IQR* Interquartile. *CAPASITS* Ambulatory Centers for Prevention and Attention for HIV/AIDS and Sexually Transmitted Infections in Spanish. Source: Authors’ creation with information of *SALVAR*

### CD4 cells and Viral Load levels over time

Table 1 also presents the data on CD4 and VL (log10) for the whole population over 2008 and 2017 and for each of the two periods of analysis (2008–2012 and 2013–2017). Overall, the number of individuals with a late diagnosis (CD4 < 200 cells/mm^3^ decreased significantly from 45% in 2008–2012 to 43% in 2013–2017 (*P* < *0.001*)). In parallel, the percentage of individuals diagnosed with a VL > 100,000 u/ml increased from 35 to 38% in the same period (*P* < *0.001*).

**Fig. 2:**
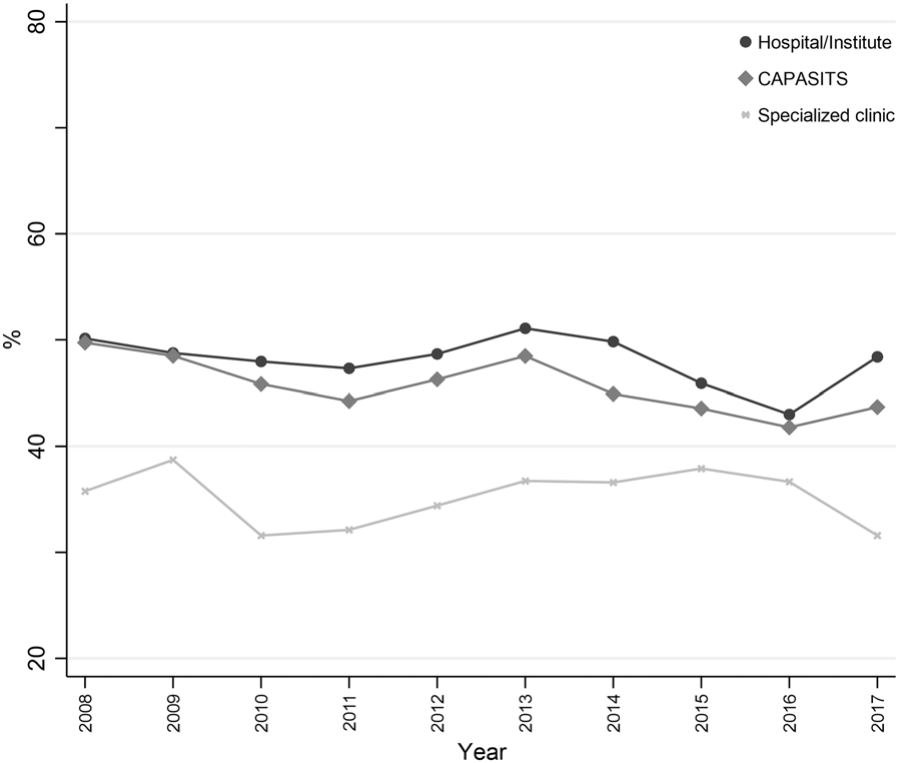
Percentage of individuals with a late diagnosis (CD4 < 200cells/mm^3^) by health facility and year. Source: Authors’ creation with information of *SALVAR*

Figure 2 presents over time the proportion of individuals diagnosed with CD4 < 200 cells/mm^3^ according to the type of facilities where they were diagnosed. Considering both periods, less individuals were diagnosed with a late diagnosis in Condesa compared to the population diagnosed in *CAPASITS* or other Hospitals/Institutes (*P* < *0.001*). More details regarding CD4 trends by year of diagnosis and region, type of health facilities, gender and age group, can be found in the Additional file 1.

### Determinants of late diagnosis

The results of the multivariable logistic regression for CD4 at diagnosis are shown in Table 2. For CD4, being diagnosed during the period 2013–2017 compared to the previous period was associated with lower odds of late diagnosis (0.96 [95% CI 0.93, 0.98]). Other factors associated with lower odds of late diagnosis were being diagnosed at *CAPASITS* facilities (0.89 [95% CI 0.86, 0.93]) or specialized clinics (0.64 [95% CI 0.60, 0.69]), compared to Hospitals. The variables that significantly increased the odds of a late diagnosis were being male compared to female (1.28 [95% CI 1.24, 1.33]), being older than 29 years old (1.85 [95% CI 1.79, 1.90], 2.24 [95% CI 1.79, 1.90], 2.423 [95% CI 2.30, 2.56], 2.34 [95% CI 2.13, 2.57] respectively for each age group), being diagnosed in a Central East (1.16 [95% CI 1.08, 1.26]) or South region (1.21 [95% CI 1.12, 1.31]) compared to Mexico City, and in a high marginalized locality (1.23 [95% CI: 1.12, 1.35]). However, as shown by the stratified analyses by time period (Table 2), being diagnosed outside Mexico City became a factor associated with lower odds during the period 2013–2017, while it increased the odds of a late diagnosis when diagnosed before 2013. The odds associated with a diagnosis at a later age were also greater for the period 2013–2017. Table 2 provides the details including the coefficients difference test across periods (Hausman test). As shown in these tables the C statistics in all models were below 0.70.

**Table 2:** Multivariate Logistic Regression for CD4 at Diagnosis, 2008–2017

Variable	OR CD4 (1: < 200 cells/mm^3^) All periods	OR CD4 (1: < 200 cells/mm^3^) 2008–2012	OR CD4 (1: < 200 cells/mm^3^) 2013–2017
Period of diagnosis (reference 2008–2012)			
2013–2017	0.96** (0.93, 0.98)		
Gender (reference female)			
Male	1.28** (1.24, 1.33)	1.33** (1.27, 1.40)	1.26** (1.20, 1.32)
Transgender	0.94 (0.77, 1.15)	1.09 (0.81, 1.47)	0.84 (0.64, 1.10)
Age group (reference 18–29)			
30–39	1.85** (1.79, 1.90)	1.66** (1.59, 1.73)	2.00** (1.92, 2.08)
40–49	2.24** (1.79, 1.90)	1.87** (1.78, 1.97)	2.59** (2.47, 2.72)
50–59	2.43** (2.30, 2.56)	1.98** (1.82, 2.1)	2.83** (2.63, 3.04)
> 60	2.34** (2.13, 2.57)	1.79** (1.56, 2.05)	2.87** (2.53, 3.25)
Region (Mexico City)			
Central East	1.16* (1.08, 1.26)	1.50** (1.34, 1.67)	0.90 (0.80, 1.01)
Central West	0.89* (0.82, 0.96)	1.16* (1.04, 1.29)	0.68** (0.61, 0.76)
Northwest	0.97 (0.89, 1.05)	1.28** (1.14, 1.43)	0.74** (0.66, 0.86)
Northeast	1.06 (0.97, 1.16)	1.49** (1.32, 1.68)	0.77** (0.68, 0.86)
South	1.21** (1.12, 1.31)	1.55** (1.39, 1.74)	0.94 (0.84, 1.05)
Type of health facility (reference hospital/National Institute)			
CAPASITS	0.89* (0.86, 0.93)	0.84** (0.80, 0.89)	0.93* (0.89, 0.99)
Specialized clinics	0.64** (0.60, 0.69)	0.70** (0.63, 0.78)	0.56** (0.50, 0.62)
Index of marginalization (reference low)			
Medium	1.07 (0.95, 1.20)	0.92 (0.77, 1.10)	1.17** (1.00, 1.37)
High	1.23** (1.12, 1.35)	0.99 (0.86, 1.15)	1.43** (1.27, 1.62)
Observations	*107,018*	*47,705*	*59,313*
C-Statistics	*0.6158*	*0.6009*	*0.6165*

Italic values indicate significance of p-value (p < 0.05)

^**^ p < 0.01, * p < 0.05, + p < 0.10. (95% Confidence Interval). OR: Odds Ratio. CAPASITS: Ambulatory Centers for Prevention and Attention for HIV/AIDS and Sexually Transmitted Infections in Spanish

## Discussion

By analyzing 10 years of data this study has provided new information to better understand the characteristics of the individuals diagnosed with HIV in Mexico between 2008 and 2017 and the impact of HIV policies implemented since 2007. Compared to the 2008–2012 period, more individuals were diagnosed at a younger age, less women were identified HIV positive, and more individuals were diagnosed in the South of Mexico in the period 2013–2017. Our univariate and multivariable analyses indicate that the actions implemented during the period 2013–17 reduced late diagnosis of HIV [17] in Mexico by 4% during that time period compared to 2008–2012. Although this difference was statistically significant the reduction is marginal and unlikely to be clinically meaningful [9]. In terms of VL, we observed in parallel an increase in the population’s levels of VL after 2013, which could be the result of the 2013–17 initiatives aimed at non-high-risk individuals (such as massive testing, increasing awareness in heterosexual populations) with an advanced stage of the disease [28]. The increase in VL between the two time periods could also be the result of the 2013–2017 policies to improve early diagnosis as some individuals may have been diagnosed with high levels of VL due to a recent infection, which is often associated with a peak, although this is an unlikely theory [29]. Still 42% of Mexicans are being diagnosed with CD4 < 200 cells/mm3, compared to a governmental objective of 30% late diagnosis in 2017. Our stratified analyses also showed a strong regional effect during the period 2013–2017, with living outside of Mexico City or being diagnosed in a CAPASITS facility decreasing the odds of a late diagnosis. These results could be explained by an increase in HIV health facilities (i.e. *CAPASITS*) outside Mexico City (from 49 in 2007 to 137 in 2017) [4, 16] and the change from a decentralized to an integrated approach to HIV treatment and care in all HIV facilities in Mexico [9] as a result of the implementation of the 2013–17 National plan.

It is difficult to compare our results to other studies conducted in Mexico due to different study designs or period of analysis. However our results are similar to the findings of Carrizosa et al. [30] who reported a late diagnosis rate of 43.2% in 2010. Findings are also aligned with the results presented by Hernandez Romieu et al.[17] who reported using data from 2007 to 2014 that being male and being older increased the odds of a late diagnosis. Compared to this study, we also showed that being diagnosed in *CAPASITS* or Condesa reduced the risk of a late diagnosis compared to being diagnosed in non-specialized HIV hospitals, which could be explained by the organization of the HIV testing centers within the Mexican Health System. As opposed to *CAPASITS* which are the main HIV treatment centers, many individuals are referred to hospitals or Institutes because they present a complicated health profile and many of them will be diagnosed with AIDS in that context. Furthermore, Mexico City has the largest hospitals and expertise to treat individuals with very advanced HIV, which could explain some of the results. Another study among women using 2012–13 data from 4 clinics in Mexico also found that being older increased the risk of late diagnosis [31]. On the other hand, while we found that being male increased the risk of a late diagnosis, a study conducted in 2011–12 in Mexico city, reported that women were more likely to be diagnosed later given that they have no suspicious of risk of infection due to their cultural and social disadvantage [16, 31]. These results could indicate that the prevention programs and early diagnosis policies no longer exclusively target high-risk populations (men who have sex with men and transgender population) and are consistent with the objectives established in the National Program in 2013 [9]. Similarly, belong to the younger group represents a protective factor for a late diagnosis, which could be explained for a higher perception of risk and, therefore routinely testing, or because the treatment is free, even unemployed and unsecured young people could access care [11, 17]. Compared to the studies previously conducted in Mexico around HIV, our study provides new information as we also evaluated the impact of the 2013 National Specific Action program. We also showed that determinants of late diagnosis changed before and after the implementation of the 2013–2017 HIV policies and found important differences at the regional level.

When interpreting the results some limitations should be noted. First it was assumed that the closest measure to diagnosis observed in *SALVAR* database for CD4 and VL was the first measure taken at moment of diagnosis of HIV, which may not be always accurate. While we conducted regression analyses to identify the determinants of late diagnosis and VL levels, we were constrained by the list of variables available in the database and there may be unmeasured confounders such as the increase in number of health facilities and distribution across Mexican regions, or some risk factors (such as no use of condom, multiple sexual partners) associated with self-perception of risk systematically differentiating the population reaching heath care. In addition, this analysis was limited by the design of SALVAR. Initially, the database was conceived to only capture data related to ART logistic, and administration. However, in 2008 (the first year of our analyses), the scope of SALVAR was expanded to incorporate and track information on CD4 and VL and other socio-economic characteristic [32], which are used in our analyses. SALVAR data base does not start collecting patient’s information when people are diagnosed, but when they start treatment; therefore, we considered that eventually those diagnosed patients will receive ART and the database will capture both dates: date of diagnosis, and date of treatment initiation. Also, while the *SALVAR* database contains a variable to document the transmission route, we could not include this variable in our models as more than 80% of the data were missing. As it is well known, transmission route is a key determinant for a self-perception of risk, and late diagnosis [33]). This could explain why our logistic regression models had moderate discrimination. Moreover, *SALVAR* does not account for the whole PLWH in Mexico, only for those registered in the Ministry of Health receiving treatment. As such, some individuals (e.g., diagnosed with an advanced AIDS-defining event that never received treatment or with a high CD4 without the need for immediate treatment) may not be registered in SALVAR. Although this proportion is unknown, we believe that this population is not large due to the existence and expansion of *CAPASITS* after 2013 and the push for a decentralized and universal access to HIV care in Mexico. Unfortunately, it is not possible to account for any correlation between testing and late diagnoses because Mexican records only capture positive diagnosis and treatment linkage. Moreover, given the design of the models, we cannot claim that policy was completely responsible for changes in levels of late diagnosis, these could be also part of an increasing awareness and readiness for HIV testing and the disease in general, and a reduction of discrimination and stigma. However, we adjusted the model by possible trend with a period variable. Finally, around 12 percent of individuals were missed as their data on date of diagnosis or CD4 measures were missing. However, the study still has a large sample size and should be representative of the PLWH receiving ART treatment in Mexico although we cannot evaluate the direction or magnitude of a possible bias regarding these missing values*.* We were also limited to the variables available in SALVAR and while several determinants associated to the health system were available for analyses, several variables which could explain late diagnosis were not available (e.g., education, distance to clinics) or had a high level of missing data (e.g., indigenous status, mode of transmission). Finally, the Mexican health public system is fragmented in two main big institutions (i.e., MoH and IMSS) and there are individuals who go from one system to another system, which means that some individuals captured in SALVAR could have been advanced on their diagnosis when changing health system. However, we believe that this should not impact the generalizability of our results as only 2.1% of individuals captured in SALVAR database would move out of the Mexican MoH system. In addition, if an individual comes from a non-SALVAR/MOH system, the key information for that patient would be transferred to SALVAR including information on date of diagnosis and CD4 measures. The study represents a first approach to associate measurable factors with late diagnosis and provide evidence for further research. Despite these limitations, our study has several strengths including the use a population-based cohort representing approximately 64% of the population receiving HIV care in Mexico. This is also the first study using and analyzing data later than 2014.

As it was mentioned before, some hypotheses can be formulated with all the information presented. We have identified key indicators, trends, and predictors of CD4 and VL levels at diagnosis to inform about the HIV/AIDS epidemic in Mexico. Although we observed a decrease in the number of individuals with a late diagnosis after the implementation of the 2013 National Specific program, this proportion is still high regarding the objective set in the 2013–2018 national plan (30%), and efforts should continue to improve HIV outcomes at diagnosis and reduce HIV transmission. Future research is also required to continue to evaluate the effectiveness of prevention programs such as increasing the HIV diagnosis rates and reaching broader populations for early detection, Furthermore, the information from SALVAR database could be analyzed to identify time from diagnosis to treatment, and what can be improved to ensure access to antiretroviral therapy through the universal access program in Mexico. Finally, additional research should be made to measure the specific differences in economic resources and possible improvements to lead changes in national programs to reduce inequalities across health facilities and Mexican regions.

## Conclusions

The results of this study indicate that the proportion of individuals with a late HIV diagnosis decreased marginally following the National HIV program implemented in 2013 in Mexico. However, the proportion of late HIV diagnosis remains much above the proposed levels in the National Specific Action Plan 2013–2018. Analyzing those factors that identify high-risk populations for a late diagnosis could expedite the achievement of policy objectives and improve national indicators of HIV in Mexico. Therefore, more efforts should be allocated to improve early detection and treatment of HIV.

## Supplementary Information

**Additional file 1.**

## Data Availability

The data that supports the findings of this study are available from SALVAR and CENSIDA Mexico, but restrictions apply to the availability of these data, which were used under license for the current study, and so are not publicly available. Data are however available from the authors upon reasonable request and with permission of SALVAR and CENSIDA Mexico.

## Abbreviations

PLWH: People living with HIV
SALVAR: Antiretroviral therapy administration, logistics, and surveillance system in Spanish.
VL: Viral Load
CAPASITS: Ambulatory Centers for Prevention and Attention for HIV/AIDS and Sexually Transmitted Infections in Spanish
SDs: Standard deviations
IQR: Interquartile
OR: Odds ratios
