# Supplementary figures and images for: Patient characteristics and determinants of CD4 at diagnosis of HIV in Mexico from 2008 to 2017: a 10-year population-based study
Source: AIDS Res Ther. 2021 Nov 13;18:84. doi: 10.1186/s12981-021-00409-0 (PMC8590317; doi:10.1186/s12981-021-00409-0)

Supplementary material.

Figure 1.

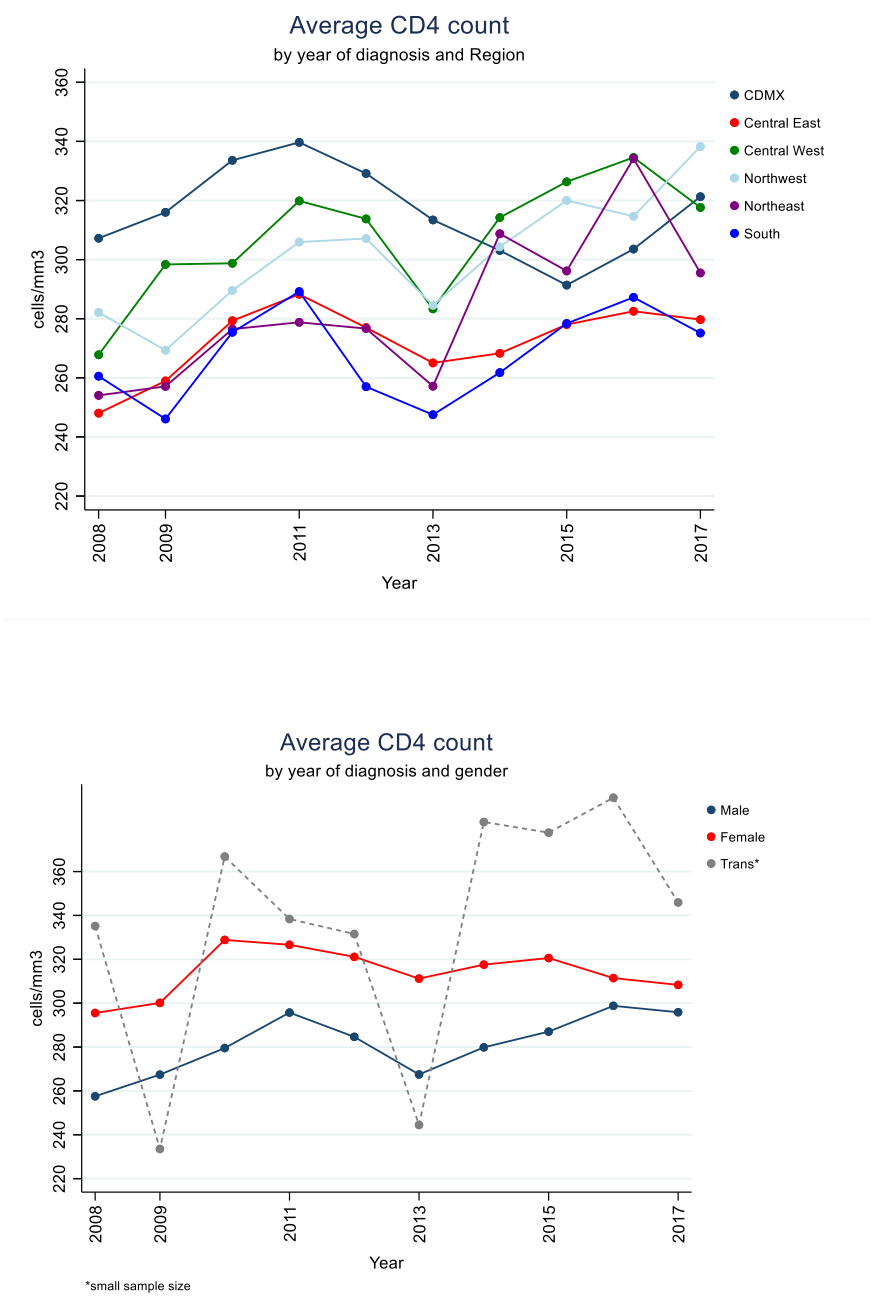

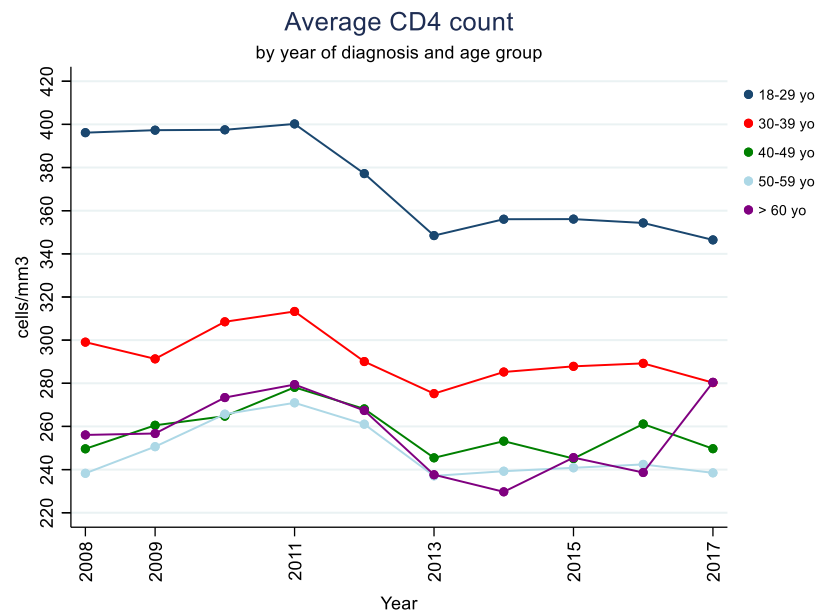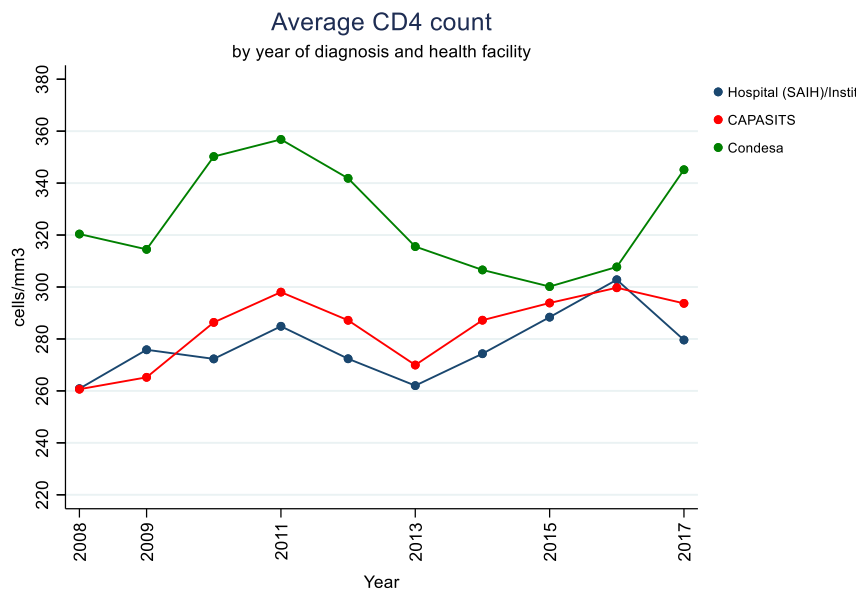

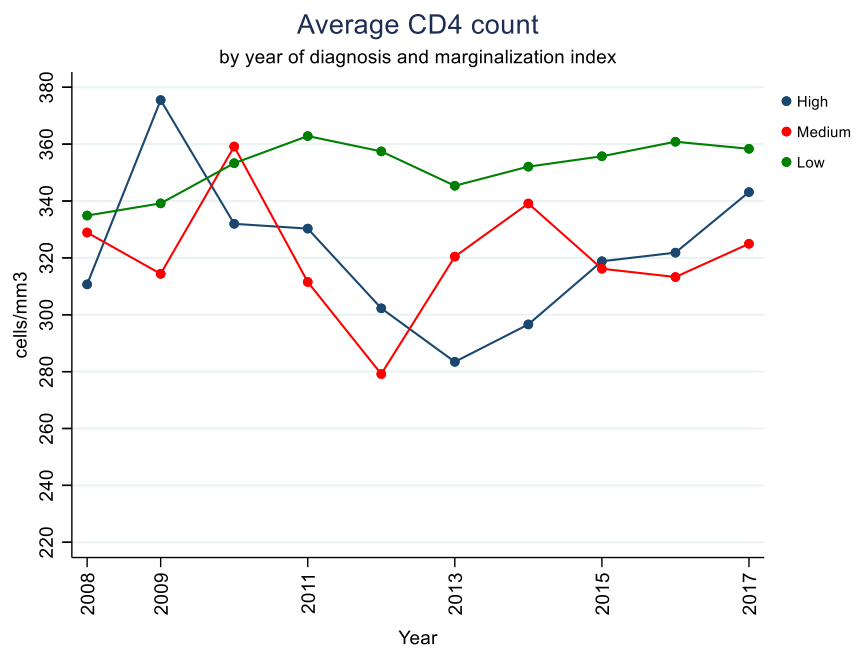

Supplement: Supplementary file 1 — Additional file 1. [file 12981_2021_409_MOESM1_ESM.pdf]
